# The effect of small-sided games with different levels of opposition on the tactical behaviour of young footballers with different levels of sport expertise

**DOI:** 10.1371/journal.pone.0190157

**Published:** 2018-01-10

**Authors:** Alba Práxedes, Alberto Moreno, Alexander Gil-Arias, Fernando Claver, Fernando Del Villar

**Affiliations:** 1 Faculty of Sport Sciences, University of Extremadura, Cáceres, Spain; 2 Sport Studies Center, Rey Juan Carlos University, Alcorcón, Madrid, Spain; Sao Paulo State University, BRAZIL

## Abstract

To optimize players' tactical abilities, coaches need to design training sessions with representative learning tasks, such as, small-sided games. Moreover, it is necessary to adapt the complexity of the tasks to the skill level of the athletes to maximally improve their perceptual, visual and attentive abilities. The objective of this study was to analyze the effect of two teaching programs, each utilizing modified games with varied levels of opposition, on decision-making and action execution in young players with different levels of sports expertise. 19 football players (U12), separated into two ability groups (Average versus Low skill-level), participated in a series of training sessions that were spread over 4 phases: Pre-intervention 1, Intervention 1 (teaching program based on modified games with numerical superiority in attack), Pre-intervention 2 and Intervention 2 (teaching program based on modified games with numerical equality). Each intervention phase lasted 14 sessions. Decision-making and the execution of pass action during league matches over the same period were evaluated using the Game Performance Evaluation Tool (GPET). The Average skill-level group showed significant differences after the first intervention in decision-making and execution of the pass action (decision-making, *p* = .015; execution, *p* = .031), but not after the second intervention (decision-making, *p* = 1.000; execution, *p* = 1.000). For the Low skill-level group, significant differences were only observed in the execution of passing between the first and last phases (*p* = .014). These findings seem to indicate that for groups with an average level of expertise, training with numerical superiority in attack provides players with more time to make better decisions and to better execute actions. However, for lower-level groups programs may take longer to facilitate improvement. Nevertheless, numerical equality did not result in improvement for either group.

## Introduction

The behaviour of football players in a competitive match depends on the action of their opponents [1]. To add complexity, players can never know with certainty what their opponents are going to do at any moment, and will have to adapt their actions with the changing game environment [2]. Although each player's actions are perceived as independent, they must also be coordinated with teammates [3]. Therefore, in a cooperative-opposition sport such as football, where open-mindedness predominates, the decision-making process is a determining factor for achieving high levels of sports expertise [4, 5].

From the perspective of ecological dynamics, decision-making is based on the interaction that an athlete maintains with the game environment [6]. In order to determine the different possibilities of action towards achieving a specific goal, and then to select a response, the athlete must engage in an active and continuous process of searching and exploring relevant information to the game context [7, 8, 9]. In this respect, the tactical behaviour of an athlete is based on intentional adaptations to the constraints imposed in a specific game situation, or during the performance of a specific task [10, 11]. Thus, to optimize players' tactical abilities, coaches need to design training sessions with representative learning tasks, i.e. tasks that ensure that practice has similar perceptual-action relationships to competitive matches [12]. Toward this goal, Small-Sided and Conditioned Games (SSCG; commonly used modified games that take place in tight spaces, involving small numbers of players and with modified rules of the game) have been proposed to be an effective methodological tool for optimizing the tactical behaviour of athletes [13, 14]. These games also promote the development of technical actions such as passing, dribbling and shooting [15], and have been shown to result in a higher level of sporting expertise in athletes since they simultaneously work on two components of action; the decision-making process and the technical execution [16, 17].

It should be noted that SSCGs are situated within the framework of Nonlinear Pedagogy (NLP) [18, 19, 20]. This new teaching-learning perspective is characterized by an integrated consideration of technical and tactical skills, and a movement away from direct instruction, a type of training does not promote the development of decision-making skills [21, 22, 23]. Nonlinear pedagogy, as part of the ecological dynamics approach, considers that in cooperative-opposition sports game actions are chosen as a consequence of the interaction between the conditions of the task and the athlete. Depending on a player's analysis of the game context they decide at each moment what to do and how to implement their selected response [24]. From this perspective, the teaching of sport focuses mainly on the manipulation of relevant constraints by simplifying game situations and guiding athletes towards reaching the objectives of the task [2, 10].

The constraints of an action may be oriented towards the athlete, towards the environment or towards the task. In team sports such as football, task-related constraints (e.g. task goals, number of players, level of opposition, space, duration, and rules of the game) are particularly relevant since they allow players to adapt their actions to a changing game environment similar to a real competitive game scenario [2, 13].

Despite the importance of tactical behaviour in the training process of young football players, most research has focused on analyzing physical and physiological parameters [25, 26, 27], and there are not too much research that study the effects of SSCGs on the game play [3, 28, 29, 30]. Within the framework of non-linear pedagogy, and more specifically in football, one of the most studied determinants of a task, and one that has been shown to change tactical behaviour, is the level of opposition. Level of opposition can be understood as the level of difficulty presented by a task due to the numerical equality or inequality of the participating teams [11, 29, 31]. In order to allow athletes in a training category to become fully competent in a particular sport, several authors have proposed that simple games where continuity is favored and that become more complex as the athlete reaches the proposed goals should be included at the beginning of the teaching-learning process [20, 32].

Investigations carried out on the match analysis in football have determined that situations of numerical inequality often occur in competitive matches [33]. It is therefore of particular relevance to analyze the impact of the manipulation of the number of partners (level of cooperation) and opponents (opposition level) in the tactical behaviour of young football players. Indeed, past investigations focused on this issue have determined that the lower the level of opposition, the lower the defensive pressure, and the more time attacking players with the ball have to make decisions, thus facilitating the process of response selection and technical execution [30, 34]. In contrast, higher defensive pressure results in lower interpersonal distance between the attacking player with the ball and the defender, and therefore less time to decide and act [30].

So, the level of sporting skills must be considered in the training planning because it influences the tactical behaviour of young football players [3, 35]. Consequently, it is necessary to adapt the complexity of the tasks to the skill level of the athletes to maximally improve their perceptual, visual and attentive abilities [20, 36]. Gonçalves, Marcelino, Torres-Ronda, Torrents, and Sampaio [33] pointed out that there is a lack of knowledge about whether the level of sporting skill influences tactical behaviour, and it is common to assess the effect of sporting skill on physical, physiological and technical variables [37], but not on variables related to the game action, such as decision-making and technical execution. Further, it is important to note that most of the studies that have attempted to analyze the effect of the level of opposition on tactical behaviour are descriptive, and no experimental investigations have been found where the effect of an intervention program based on the manipulation of the number of colleagues and/or adversaries on decision-making and technical execution in football has been analyzed, and neither keeping in mind the level of sporting skills.

The main objective of the study was therefore to analyze the effect of two training programs, each based on modified games with different level of opposition, on decision-making and technical execution in two groups of young football players of different abilities.

## Methods

### Participants

The participants were 19 football players from the under-12 category of two teams from the same Spanish club. The participants were part of two teams that were previously formed by the club for competition. Both teams had different levels of sports expertise and participated in different leagues comprising teams of appropriate skill levels. The Average skill-level group consisted of 10 players (age, M = 10.55, SD = 0.51; years of experience, M = 3.9, SD = 1.19), while the Low skill-level group consisted of 9 players (age, M = 10.66, SD = 0.5, years of experience, M = 3.11, SD = 1.45). Each group had the same amount of training: two weekly sessions of one hour each one. Players who didn´t play more than one game in each phase were not being consider in this study. A homogeneity analysis was carried out in pre-intervention phase 1 and significant differences were observed for both decision-making (Levene's statistic = 13.989, p = .002) and the execution of the pass action (Levene´s statistic = 10.341, p = .005).

The research has been developed under the recommendations of the Declaration of Helsinki. The participants and their parents were informed of the study. As the participants were under 18, the parents signed an informed consent. The research project was fully approved by the Ethics Research Committee of the University of Extremadura (Spain).

### Procedures

This is a quasi-experimental design with two groups (Average skill-level group and Low skill-level group), carried out in four research phases (see Table 1). For this, to each group, a pre-post design was used in order to assess the effects of two intervention programs on each group.

**Table 1:** Schematic of the study design and schedule.

Season 2015/2016				
October	November-December	Christmas Holidays	January	February-March
Pre 1	Intervention 1		Pre 2	Intervention 2
3 sessions (3 matches)	14 sessions (7 matches)		3 sessions (3 matches)	14 sessions (7 matches)

To do that, the execution and the decision-making in the pass actions were evaluated in all matches of the league (both matches, played as local or as visitant). All of participants of this study played all of them. Each match had a length of 48 minutes. It is important to highlight that when it concerns about footballers in formative stages, all of them play the same time. However, the goalkeeper was not analyzed. The four research phases are explained below:

#### Pre-intervention 1

To understand the initial level of each group prior to the first intervention, decision-making and execution were recorded and registered for the players in the first three matches of the league, obtaining the mean of each variable of the three matches. In this phase, consisting of 6 sessions, the coach conducted his training sessions following the model of direct instruction, an approach characterized by decontextualization and unlike Nonlinear Pedagogy. In this phase, each team faced rivals with the same level of expertise, thus controlling the level of opposition.

#### Intervention 1

In this phase, the first program of teaching based on modified games in numerical superiority in attack was applied. This program consisted of a total of 14 training sessions, with 2 sessions lasting one hour each per week. The intervention program was identical for both groups. There was also a follow-up and detailed observation of the development of training to ensure that the intervention program was being implemented correctly. At the same time, the seven matches played as part of the regular league were recorded and registered so that decision-making and execution could be observed.

#### Pre-intervention 2

This phase was carried out following the same procedure as Pre-intervention phase I, and for the same purpose: to establish the initial level prior to the second intervention. Once again, the level of opposition for the matches played in this phase was the equal to that of the team.

#### Intervention 2

In this phase, unlike Intervention 1, a teaching program based on games modified in numerical equality was applied. All other procedural aspects were the same.

### Variables

#### Independent variables

The study considered two independent variables: the level of opposition and the level of sports expertise of the participants. The level of opposition is understood as the level of difficulty that the task presents due to the numerical equality or inequality of the participating teams [31]. The level of sport expertise such as the result of the successful interaction between biological, psychological, and social factors [38].

In relation to the opposition level, two training programs were developed under the NLP approach: one based on modified games using numerical superiority in attack, and another based on modified games with numerical equality. Both programs were conducted in both groups across 14 football training sessions during seven weeks (two weekly sessions of one hour each one). The objectives for the sessions of both group, in which an integration of technical and tactical aspects was always sought, are displayed in Table 2.

**Table 2:** Scheme of work used in the study in each intervention phase.

Session number	Session objectives	
	Attack	Defense
1	Space (width and depth in attack)	Prevent lines of passes and anticipation
2	Penetration (attack the goal)	Covering
3	Mobility to interchange of positions	Pressing
4	Dealing with crosses	Closing down
5	Mobility to create lines of pass	Balance (cut lines of passes)
6	Creation and occupation free spaces	Marking
7	Penetration (creation of an advantage in number)	Occupy spaces
8	Space (width and depth in attack) II	Prevent lines of passes and anticipation II
9	Penetration (attack the goal) II	Covering II
10	Mobility to interchange of positions II	Pressing II
11	Dealing with crosses II	Closing down II
12	Mobility to create lines of pass II	Balance (cut lines of passes) II
13	Creation and occupation free spaces II	Marking II
14	Penetration (creation of an advantage in number) II	Occupy spaces II

All training sessions were based mainly on the NLP pedagogical principles of representation, tactical complexity and exaggeration. The following explains how each of these was implemented:

- *Representation*: In each training session there were 4 modified games of 15 minutes each, characterized by situations similar to those experienced in a real game context, but in a simplified form. For this, the number of players (from 2 to 5 players per team) and the space (between 30x15m and 40x25m) were reduced. This allowed the player to have more frequent contact with the ball [39] and thus favoured the development of technical skills [40]. More specifically, numerical superiority tasks were proposed in the following format: 3 vs 2 (in 30x15m); 4 vs 3 (in 35x20m) or 5 vs 4 (in 40x25m); the tasks of numerical equality were either 3 vs 3, 4 vs 4 or 5 vs 5.
- *Tactical complexity*: In order to give the young players more time to make decisions, the first teaching program was based on modified games with numerical superiority in attack (the number of opponents was one less than that of the attackers, e.g. 5 vs. 4, 4 vs. 3 or 3 vs. 2). To do that, there was usually a wildcard in the game (e.g. 4 vs. 4 + 1 wildcard) or one player of the team that hasn’t the ball possession doesn’t´ play (e.g. he was waiting behind the goal). The second program was based on games with numerical equality. In all tasks, the number of players was reduced in order to adapt its complexity to the ability level of the athletes.
- *Exaggeration*: For each task, the rules of the game were manipulated in order to emphasize the tactical learning objective (e.g. in a 4 vs. 3, or 4 vs. 4, if depth in attack was the learning objective a line was drawn and players were instructed that in order to pass to the next zone, it had to be done receiving a pass while running). This principle was also present in the reward system of the task, increasing the score of the team that achieved it (e.g. passing the ball to the second zone from the opposing side meant giving more value to the orientation changes).

In order to guarantee the correct application of the teaching program, the coach was instructed by an expert. The expert was a professor in Sport and Exercise Sciences and has 12 years´ experience in football at young stages. As in the previous studies [41, 42, 43], the training program to instruct the coach was developed over three weeks that the pre-intervention lasted. In the first week, the coach was required to read three NLP-related articles [3, 44, 45]. For each article, the coach met with the first author to discuss the contents. In the second week, the coach designed a series of tasks based on the principles of NLP. Finally, in the third week a practical application of the tasks took place with football team of the same age category as the participants of the present study.

To ensure that the model was correctly applied [46], the training sessions were supervised by a researcher with 15 years’ experience supervising teaching methodologies and he also attended the training sessions. A 11-item checklist (see Table 3) was adapted to test the behavioural fidelity of the coach according to the NLP. This researcher and with the first author randomly selected sessions for the assessment of the presence or absence of the items included in Table 3. A sample of 5 sessions for each intervention was observed, more than 10% the total sample [47]. A 100% agreement was reached between the two observers, who confirmed that all key aspects included in the checklist with regard to the features of the NLP were used in each observed-session.

**Table 3:** Instructional checklist.

Date:	Present	Absent
1. All the tasks are related to small-sided games. 2. Modifications to the full-game were performed. 3. All the tasks have different solutions. 4. The coach simply explains the task without providing solutions. 5. The number of players per team is between 2 and 5. 6. The pitch is reduced proportionally to the number of players. 7. The defense always has an active role. 8. The numbers of touches are not limited for any task. 9. In Intervention 1 there is a numerical superiority in attack for all tasks. 10. In Intervention 2 there is a numerical equality for all tasks.		

The level of sporting skill of the teams was determined according to their category designation. In non-professional football clubs in Spain, at each age level teams are configured according to their level of expertise (e.g., A team, B team, C team, etc.) [48]. The aim of this is to form homogeneous teams as far as players are concerned, and for teams to compete in leagues with teams of an equal level. Based on this, in the present study one team was characterized as having an average level of skill, and the other as having a low level of skill since they participated in different leagues.

### Dependent variables

Dependent variables of this study were decision-making and execution. Decision-making as the process whereby athletes select one type of attack from a series of alternatives to execute it at a specific moment and in a real game situation [49]. It was measured by the percentage of successful decision over the total number of decisions made. Execution is defined as the performance, outcome, or the final result of the motor execution [49]. It was also measured by the percentage of successful execution over the total number of execution made.

### Instrument

The decision-making and execution assessment was based on indirect and external systematic observation, a methodology that had been used in previous studies to measure athletes’ decision-making and execution in real game situations [50]. To assess the decision-making and execution of football players, the GPET observation instrument [51] was used. This instrument, that it had already been used for other studies in young football [4, 17 43], is an adaptation to football of the original “Game Performance Assessment Instrument (GPAI)” [52] which was created to assess performance in the game, from a sporting tactic viewpoint. This instrument permitted evaluating the player’s tactical problem-solving skills, by means of selecting and applying an appropriate technique, and evaluating both measurements (decision-making and execution) in real game situations, as recommended by [53].

All the pass actions of each one of the players on the team were recorded. To evaluate decision-making, the *decision-making* component of this instrument was used, assigning value 1 to appropriate decisions and with a 0 to inappropriate decisions. Likewise, to evaluate execution, the *execution* component of the same instrument was used, assigning value 1 to successful executions and unsuccessful executions with a 0 (see Table 3). With respect to the criteria proposed to assess the decision-making and the execution, it must be mentioned that, due to the actual characteristics of the instruments, all the criteria were equally important and therefore, there was no type of hierarchy. This percentage of successful decisions was calculated individually for each participant. To calculate the percentage of successful decisions and executions, the total number of these decisions and executions was divided by the sum of the number of the total of decisions and executions and multiplied by 100 [23]. The criteria that were considered to assess if the decision and execution taken were successful or unsuccessful are specified in Table 4.

**Table 4:** GPET coding procedures for decision-making and execution in the pass action (football) (García-López et al., 2013).

PASS ACTION	
Decision-making	1	- Passing to a teammate who is unmarked.
	0	- Passing to a player who is marked closely or there is a defensive player in a position to cut off the pass. - Passing to an area of the pitch where no team-mate is positioned.
Execution	1	- Successful pass to a teammate: to his body if he is stationary, lead pass if he is running. - Appropriate length and speed.
	0	- Interception. - Pass is too hard. Out of play. - Pass is too far behind or in front of a teammate.

A total of 4901 passes (pre 1, n = 772; int 1 = 1660; pre 2 = 775; int 2 = 1694) were observed (Average skill-level group, n = 2474, Low skill-level group, n = 2427), across the 20 matches of the Extremadura football league of the 2015/2016 season. All the matches were recorded using a Sony HDRXR155 camera, from a fixed position, using a Hama Gamma Series. The camera was always placed in the background of the playing field, at a height of 4 meters, guaranteeing an optimal view of all the game actions.

After that, decision-making and execution were analyzed for each action. The values were registered in an Excel worksheet and then, they were moved to the SPSS program to develop the statistical analysis.

With respect to the inter-observer reliability, two research observers were trained to analyze decision-making and the execution of pass action. These observers were trained by an expert in football (Level 1 by the Spanish Football Federation), who has 4 years of experience in observational methodology (researcher with experience in research projects).

As a preliminary step, the expert met with the observers to clarify possible doubts about the observation instrument and the coding criteria of each dependent variable (decision-making and execution) for the pass action. Subsequently, the observations were carried out, and 510 passages were analyzed, a sample of more than 10% of the total [47]. Inter-observer reliability was calculated using the following formula: agreements/(agreements + disagreements) x 100 measure. Once this value was calculated, the Cohen kappa index was used. All training values were observed to be above .90, surpassing the value .81 from which an adequate agreement is considered [54], thus achieving the necessary reliability for the subsequent coding of the dependent variables.

To guarantee the time reliability of the measurement, the same sample of matches was analyzed with a time difference of ten days, obtaining intraobserver reliability results of .92. These results reflected very good concordance, thus obtaining the necessary reliability for the subsequent coding of the dependent variable.

### Data analysis

The statistical program SPSS v21.0 (Chicago, IL) was used for the data analysis and processing. Data normality was examined through the Shapiro-Wilks test, indicated data normality, which led to the use of parametric statistics. To compare the mean scores of each group in the different dependent variables, a repeated measures analysis of variance, MANOVA 2x2 (Test-Time x Group) was carried out. The four phases of the study (pre 1, int 1, pre 2 and int 2) were considered in the repeated measures factor, whilst both groups (average skill-level and low skill-level) were included in the group factor. Analysis of differences was performed by means of multivariate contrasts, which are reported in this type of analysis. Effect sizes were calculated using the partial eta-squared statistic (ηp2), as this allowed us to know the extent of the differences found, on minimizing the influence of the sample size. The level of statistical significance was established for p ≤ .05, with a confidence interval for differences of 95%.

## Results

In the intra-group analysis, the multivariate contrasts showed that there were significant differences in the Average skill-level group between the four measurements carried out in the research (Λ Wilks = .301; F(6, 12) = 4.634; p = .012; η_*p*_^2^ = .699; SP = .890). With respect to the Low skill-level group, the multivariate contrasts showed significant differences between the four measurements carried out in the research (Λ Wilks = .219; F(6, 12) = 7.134; p = .002; η_*p*_^2^ = .781; SP = .982). The comparisons in pairs between the different phases of the study are then presented for each of the groups.

For the Average skill-level group (Table 5), both decision-making and execution were found to have significant differences between the Pre-intervention 1 and Intervention 1 phases, and between the Pre-intervention 1 and Pre-intervention 2 phases. No others differences were found.

**Table 5:** Descriptive statistics and pairwise comparison of the decision-making and the execution of the pass between the different measures. Average skill-level group.

Mea-sure	Time (I)			Time (J)			Mean difference (I-J)	Typical error	*P*	*IC 95% diferencias*	
	T´	M	SD	T´	M	SD				L.L	UL
DM	Pre1	.706	.054	Int1	.843	.039	-.138	.039	.015	-.253	-.022
	Pre1	.706	.054	Pre2	.886	.062	-.180	.039	.002	-.297	-.063
	Pre1	.706	.054	Int2	.838	.054	-.132	.074	.074	-.274	.009
	Int1	.843	.039	Pre2	.886	.062	-.043	.033	1.000	-.141	.056
	Int1	.843	.039	Int2	.838	.054	.005	.028	1.000	-078	.088
	Pre2	.886	.062	Int2	.838	.054	.048	.035	1.000	-.056	.152
EX	Pre1	.593	.064	Int1	.714	.052	-.121	.038	.031	-.234	-.008
	Pre1	.593	.064	Pre2	.743	.085	-.150	.044	.020	-.282	-.019
	Pre1	.593	.064	Int2	.697	.059	-.104	.045	.192	-.237	.029
	Int1	.714	.052	Pre2	.743	.085	-.029	.035	1.000	-.132	.074
	Int1	.714	.052	Int2	.697	.059	.017	.031	1.000	-.075	.109
	Pre2	.743	.085	Int2	.697	.059	0.46	.038	1.000	-.066	.159

Note. M = mean; SD = standard deviation; DM: Decision-making; EX: Execution; T´: Time; Pre 1: 1^st^ pre-intervention phase; Int 1: 1^st^ intervention phase; Pre 2: 2^nd^ pre-intervention phase; Int 2: 2^nd^ intervention phase; I: first time; J: second time; CI: confidence interval; LL: lower limit; UL: upper limit.

For the Low skill-level group (Table 6), the only significant difference observed was for the execution variable between the Pre-intervention 1 and Intervention 2 phases.

**Table 6:** Descriptive statistics and pairwise comparison of the decision-making and the execution of the pass between the different measures. Low skill-level group.

Mea-sure	Time (I)			Time (J)			Mean difference (I-J)	Typical error	*P*	*Differences 95% CI*	
	T´	M	SD	T´	M	SD				LL	UL
DM	Pre1	.586	.225	Int1	.655	.124	-.069	.041	.645	-.191	.052
	Pre1	.586	.225	Pre2	.645	.174	-.059	.041	1.000	-.182	.064
	Pre1	.586	.225	Int2	.650	.082	-.064	.050	1.000	-.213	.084
	Int1	.655	.124	Pre2	.645	.174	.010	.035	1.000	-.094	.115
	Int1	.655	.124	Int2	.650	.082	.005	.029	1.000	-.083	.093
	Pre2	.645	.174	Int2	.650	.082	-.005	.037	1.000	-.115	.104
EX	Pre1	.483	.201	Int1	.560	.138	-.077	.040	.412	-.196	0.41
	Pre1	.483	.201	Pre2	.555	.154	-.072	.047	.834	-.211	.067
	Pre1	.483	.201	Int2	.650	.082	-.168	.047	.014	-.308	-.027
	Int1	.560	.138	Pre2	.555	.154	.005	.036	1.000	-.103	.114
	Int1	.560	.138	Int2	.650	.082	-.090	.032	.078	-.187	.007
	Pre2	.555	.154	Int2	.650	.082	-.095	.040	.172	-.214	.024

Note. M = mean; SD = standard deviation; DM: Decision-making; EX: Execution; T´: Time; Pre 1: 1^st^ pre-intervention phase; Int 1: 1^st^ intervention phase; Pre 2: 2^nd^ pre-intervention phase; Int 2: 2^nd^ intervention phase; I: first time; J: second time; CI: confidence interval; LL: lower limit; UL: upper limit.

## Discussion

The objective of this study was to analyze the effect of two teaching programs, each utilizing modified games with different levels of opposition, on decision-making and execution in young players with different levels of sports expertise. In the following section the results will be discussed according to the independent variable "level of opposition".

### Intervention program based on modified games in numerical superiority in attack

Concerning the Average skill-level group, the results show significant differences in both decision-making and the execution of passes after an intervention program based on modified games with numerical superiority in attack. These findings indicate that this teaching program was effective for the pass action, allowing an improvement of decision-making and execution in the players of the Average skill-level group. Therefore, the results obtained for this group indicate that in the design of training tasks, manipulating the level of opposition by reducing the number of opponents is an effective strategy for amplifying the sources of information that regulate decision-making [9, 29] and therefore, to favor the development of both cognitive and performance variables. In this sense, the representativeness of the practice through SSCG allowed the students to train in a changing learning environment, with a constant uncertainty characteristic of the competition parties [19]. In addition, the great variety of training tasks gave players a great diversity of practice situations and perception-action patterns, which can be argued to have promoted a constant exploration and creativity on the part of the player [55].

In contrast, for the Low skill-level group the results do not indicate the same differences after the first phase of intervention for any of the variables studied. These results thus highlight the need to assess the level of expertise of players in order to ensure that constraint modifications made in modified games are done in the most appropriate direction.

The fact that this group did not obtain significant differences after the intervention program may be explained by the length of intervention programs (i.e. the 7 weeks with the 14 training sessions may be was not enough). Faubert [56] points out that more skilled athletes learn faster than those who are less skilled. It is important to note, however, that the weaker group had the same amount of training as the average skill group. For these players to improve, they may need more time training with numerical superiority in attack. In this regard, Verburgh, Scherder, Van Lange, and Oosterlaan [57] showed that in the learning process of a certain movement pattern, the learning phase is followed by consolidation, and it is possible that the players of the Low skill-level group did not reach this second phase.

Another possible explanation for the results from the Low skill-level group may be related to the complexity of the tasks, which may have been high for these players. Ayvazo and Ward [58] point out that coaches should consider that players need an affordable challenge for learning when designing training tasks. Perhaps this group would have made more improvement if they had been trained with tasks where there was more numerical superiority (e.g. 3 vs. 1 or 4 vs. 2). In addition, Tan and colleagues [20] pointed out that this type of practice could be complemented by the practice of modified games of exaggeration. These authors suggest that the design of this modified game typology promotes optimal learning environments. Because it has been shown that players with a higher level of skill present better perceptual abilities [36], for more inexperienced players the manipulation of constraints such as the rules of the game (e.g. objective: receive deep pass behind the defense; rule: place a dashed line and enforce the rule that in order to move to the next area, the player must be receiving a pass while running), can be an effective tool to facilitate learning [33].

### Program of intervention based on games modified in numerical equality

Unlike the first intervention program, for the second intervention, which was based on numerical equality, identical results were found for both groups: No significant improvements were observed for decision-making or for the execution of passes.

Firstly, one might think that since there is normally a numerical equality in attack during competitions it would be better for training sessions to be the same in order for players to experience the same level of defensive pressure. However, our results are not consistent with this. One explanation could be that in the training sessions, and referring specifically to youth teams, one of the objectives is the assimilation and learning of new concepts [57]. Thus it may be essential to practice tasks with less tactical complexity than a real context. For the Average skill-level group, it may be that the lower level of opposition in the first intervention phase (numerical superiority in attack) has simplified perceptual and action skills, and thus facilitated the process of learning [13, 53] and enhanced implicit learning without the need for explicit instructions [20]. Conversely, the numerical equality of the tasks in the second phase may have impeded this learning process, both for the Average and Low skill-level groups.

Recently, research has focused on assessing the effects of the level of opposition in modified games, frequently obtaining favorable results when there was numerical superiority in attack. Specifically, Sampaio and colleagues [31] and Travassos and colleagues [11] observed that when the number of attackers was higher than the number of defenders, the lowered defensive pressure facilitated the successful execution of skills. In the second intervention phase of this experiment, it is possible that the numerical equality prevented the learning of these skills. On the other hand, Castellano, Silva, Usabiaga, and Barreira [59] observed that the presence of wildcards in the attack resulted in a decrease of errors in the pass action, due to the numerical superiority that existed in the attack phase. Thus, the greater participation of the players, facilitated by a continuity in the game, allowed a greater learning of technical-tactical actions. According to the postulates of the theory of deliberate practice, this is because there is a significant and positive relationship between practice and performance [60].

Consequently, while for the Low skill-level group it is necessary to use modified games with greater numerical superiority in attack (e.g. 3 vs. 1 or 4 vs. 2), for the Average skill-level group to improve tactical concepts and technical skills, coaches must implement tasks with a lower level of complexity than experienced in competition, designing tasks where there is numerical superiority in attack. In this way, the players will have more time to make decisions and, consequently, to improvement in both cognitive and performance variables [43].

One of the limitations of the study was the number of participants, which limits the capacity to extrapolate the results. The research was conducted with all team´s players, so an increase in sample would require a design with more participating teams and coaches, which may present issues of experimental control in the intervention phase. We must highlight that, in order to maintain the ecological validity, the study was developed in a natural context, in which comes about an unequal number of game actions per player.

## Conclusions and practical applications

In the process of training footballers, when it comes to planning objectives and contents to teach, and then designing learning tasks, we have highlighted that it is important to consider the athletes’ level of expertise. In this regard, the findings obtained in the present study indicate that using numerical equality during training is not effective for improving decision-making or skill execution. Further, for players with an average level of sporting skill, developing modified games in which there is numerical superiority in attack is considered essential. Finally, for players with a low level of sporting skill, our findings suggest that it is necessary to favor situations of less tactical complexity than those proposed in the study (e.g. 3 vs. 1 or 4 vs. 2), in order to favor adequate learning.

## Supporting information

S1 File Data analysis. (SAV)
